# Reactivity and Bond Strength of Universal Dental Adhesives with Co-Cr Alloy and Zirconia

**DOI:** 10.3390/dj7030078

**Published:** 2019-08-01

**Authors:** Dimitris Papadogiannis, Maria Dimitriadi, Maria Zafiropoulou, Maria-Dimitra Gaintantzopoulou, George Eliades

**Affiliations:** Department of Biomaterials, School of Dentistry, National and Kapodistrian University of Athens, 115 27 Athens, Greece

**Keywords:** universal adhesives, reactivity, bond strength, Co-Cr alloy, zirconia

## Abstract

The aim of this study was to evaluate (a) the reactivity of six universal dental adhesives with polished cobalt-chrome (Co-Cr) alloy and zirconia (3Y-TZP) surfaces; and (b) to assess the shear bond strength (SBS) of a resin composite with polished and alumina-blasted surfaces as mediated by these adhesives. The products tested were Adhese Universal (AD), All-Bond Universal (AB), Clearfill Universal Bond (CB), G-Premio Bond (GP), Prelude One (PO) and Scotchbond Universal (SB). The reactivity on polished substrates was evaluated by reflection infrared microscopy (RFTIRM). The roughness parameters of polished and 50 μm alumina grit-blasted surfaces were assessed by optical profilometry. The SBS of the composite bonded to the substrates treated with each adhesive (n = 10/product) was evaluated after 1 week of storage (H_2_O/37 °C) by Weibull statistics. Evidence of phosphate interaction with polished substrates was obtained by FTIRM, with higher peaks on the alloy. Alumina-blasting increased all roughness parameters with higher values on the alloy. AD, CB were the strongest (*σ*_0_) treatments on alloy surfaces and AD, CB, AB, SB on zirconia. GP was the weakest on both substrates and the least reliable (*β*) on alloy. On polished alloy GP, PO performed better (*σ*_0_), whereas on zirconia there were no significant differences. All adhesives showed more prominent reaction with the Co-Cr alloy than with 3Y-TZP.

## 1. Introduction

Universal or multi-mode dental adhesives, the most recent development in the field, are blends of adhesive monomers of mild to moderate acidity, at lower concentration than their “etch and rinse” precursor adhesives, with conventional hydrophobic crosslinking monomers, acid-compatible catalysts and properly selected solvents to provide thin and homogeneous adhesive films [1]. Their main goal is to offer flexibility in the choice of treatment protocols for various substrates. Designed with the “all-in-one” concept introduced by one-step self-etch adhesives [2], they can adapt to each clinical situation, giving freedom to the clinician [3], while saving time and the need for multiple products in various applications. The universal adhesives, apart from bonding to dental hard tissues, are indicated for bonding to glass-ceramics, polycrystalline ceramics and metals [4], including a wide spectrum of intraoral repairs [4,5].

Although a significant amount of laboratory testing has been performed on universal adhesives, the majority is focused on the bond strength of these materials with enamel [5,6,7] and dentin [1,8,9] under different treatment strategies (etch-and-rinse, self-etch) [10], the interfacial sealing capacity and the fatigue resistance [11,12]. Some studies have been performed on zirconia [13,14,15], considering the long-term reliability problems of adhesive bonding to this material [3], and very few studies have been carried out on metals, mainly for resin veneering of paediatric stainless steel crowns [16,17] and bonding to noble alloys [18].

The aim of this study was to evaluate the reactivity of various commercially available universal dental adhesives with polished cobalt-chrome (Co-Cr) alloy and zirconia surfaces and to evaluate the bond strength of a resin composite to the two substrates by these adhesives before and after grit-blasting. The null hypothesis was that no significant differences would be found in the bonding mechanism on Co-Cr and zirconia and that grit-blasting does not improve bond strength.

## 2. Materials and Methods

Six commercially available universal dental adhesives were tested in this study (Table 1). All the adhesives contain the 10-methacryloyloxydecyl dihydrogen phosphate adhesive monomer (10-MDP), four products (AD, GP, PO, SB) contain carboxylic/polycarboxylic monomers, two (CB, SB) silane and one (GP) a thiophosphate adhesive monomer. The properties tested were (a) the reactivity of the adhesives with a Co-Cr alloy and zirconia (3% mol yttria stabilized tetragonal zirconia polycrystals or 3Y-TZP); (b) the roughness parameters of these substrates before and after alumina-blasting; and (c) the interfacial strength of a resin composite restorative to these substrates as mediated by the adhesives.

### 2.1. Reactivity with the Substrates 

Cast disc shaped specimens of a Co-Cr alloy (Girobond NBS, Amann Gierrbach GmbH, Pforzheim, Germany) and computer-aided design/computer-aided manufacturing (CAD/CAM) 3Y-TZP specimens (Lava, 3M ESPE, Seefeld, Germany) were fabricated (Ø = 7mm, h = 2mm), wet-ground/polished up to 2000 grit-size SiC papers in a polishing machine (Dap-V, Struers, Ballerup, Denmark), ultrasonicated for 5 min in water, immersed in ethanol (2 min) and air-dried (20 s). Spectra of the cleaned alloy and zirconia surfaces were obtained by reflection Fourier transform infrared microscopy (RFTIRM), employing an FTIR microscope (AutoImage, Perkin-Elmer, Buckinghamshire, UK) equipped with a liquid-N_2_ cooled mid-band mercury-cadmium telluride detector, under the following conditions: 4000–650 cm^−1^ wavenumber range, 4 cm^−1^ resolution, 400 × 300 μm^2^ aperture and 300 scans co-addition. The surfaces were then treated with each adhesive according to the manufacturers’ instructions. The films formed were kept intact for 15 min (dark/dry conditions at 37 °C) and then were rinsed off with water (10 mL) and acetone (5 mL) to remove the physisorbed fraction of the adhesives from the chemisorbed one, and RFTIRM spectra were taken again as before, by focusing on regions with residual adhesive. All reflectance spectra were converted to absorbance by Kramers–Kroning transformation. Transmission spectra of the uncured adhesive films brushed on acid-resistant Ge windows and air-dried to remove solvents were plotted in absorbance scale and used as controls. The experiment was replicated in triplicate for each adhesive/substrate combination.

### 2.2. Roughness

Specimens of alloy and zirconia were prepared as before (n = 20 per adhesive and substrate). Half the specimens were kept intact (group A), whereas the rest were blasted with 50 μm α-alumina powder (corundum) under the same conditions using an intraoral etcher (Microetcher ΙΙA, Danville Materials, S. Ramon, CA, USA) operated under 0.23 MPa air-pressure at 5 mm distance and 90° angle for 5 s (group B). The alumina-blasted substrate surfaces were cleaned with a strong stream of dry air for 20 s. Five randomly selected specimens from each group were used to determine the surface roughness employing an optical profiler (Wyko NT 1100, Veeco, Tuscon, AZ, USA) operated under vertical scanning imaging mode, 2 mm scanning length, 40× magnification, 113.3 × 148.5 μm^2^ analysis area, 2% modulation and tilt correction with 0.1 nm (z-axis) and 0.2 μm (x-, y-axis) resolution. The parameters measured were the Sa, Sz (amplitude), Sdr (hybrid) and Sc, Sv (functional). Sa is the arithmetic average of the absolute values of the surface height deviations measured from the best fitting plane; Sz is the 10 point height over the surface, representing the average difference between the five highest peaks and five lowest valleys; Sdr is the developed area due to the surface texture versus an ideal plane area ratio, Sc (core void volume) is the volume supported by the surface from 10–80% of the bearing ratio and Sv (surface void volume) is the volume the surface would support from 80% to 100% of the bearing ratio. On each specimen three measurements were performed and averaged to yield a representative value.

### 2.3. Bond Strength

All the specimen surfaces were covered by an adhesive tape of 100 μm thickness with a hole of a 3 mm in diameter, located at the center of each specimen. For each adhesive, 10 specimens were randomly selected and subjected to the priming treatments according to the manufacturers’ information. The adhesives were light-cured for 15 s with a LED curing unit (Radii Plus, SDI, Bainswater, Victoria, Australia, 1.5 W/cm^2^ intensity at standard high intensity mode). Acetal molds of 3.5 mm internal diameter and 2 mm height were placed over the treated areas, filled with a resin composite (Tetric Evoceram, Ivoclar-Vivadent, Schaan, Liectenstein) in two 1 mm increments and light-cured for 20 s each. All the specimens were stored in water for 1 week at 37 °C and then subjected to shear loading at the substrate-composite interface with the notched-edge blade method [18] in a universal testing machine (Tensometer 10, Monsanto, Swidon, UK) operated at a crosshead speed of 0.5 mm/min. The debonded surfaces both for the alloy and zirconia specimens were examined at 10× magnification under a stereomicroscope (M80, Leica, Wetzlar, Germany) to characterize the failure mode (Type I: Adhesive failure, Type II: Cohesive resin failure and Type III: Mixed type I and II failure).

### 2.4. Statistical Analysis

The distribution of the roughness parameters results did not pass in all cases normality and equal variance tests, hence in these cases a non-parametric Mann–Whitney test was used instead of a *t*-test for statistical comparisons. The SBS data were statistically evaluated by Weibull analysis. The shape or modulus parameter*-β* (variability of the results by expressing the size distribution of the flaws), the scale or B63.2 parameter*-σ*_0_, (characteristic life, by indicating the strength value for which the 63.2% of the sample size were debonded) and the strength at 5% failure probability (*σ*_0.05_) of the Weibull distributions were calculated. Failure mode was evaluated by a Chi-square test. The statistical analyses for roughness, and failure mode were performed by GraphPad Prism software (Graph Pad Software, San Diego, CA, USA). For the Weibull analysis the OriginLab software (v9.1 SRO, Northampton, MA, USA) was used. For all cases, a 95% confidence level was selected (α = 0.05). 

## 3. Results

**Figure 1:**
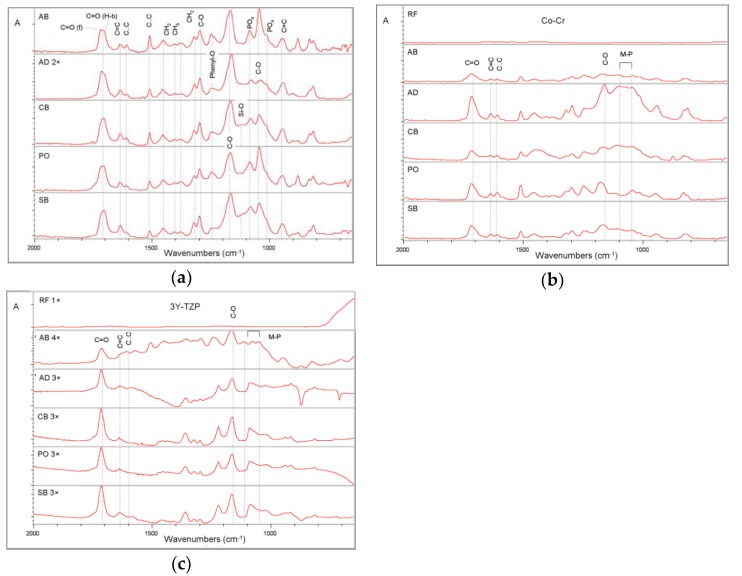
(**a**) Transmission FTIR spectra of reference adhesives, (**b**) Reflection infrared microscopy (RFTIRM) spectra of reference cobalt-chrome (Co-Cr) alloy before (RF) and after the adhesive treatments plus water and acetone rinsing, and (**c**) RFTIRM spectra of reference zirconia (3Y-TZP) before (RF) and after the adhesive treatments plus water and acetone rinsing. Note the absence of peaks from reference Co-Cr and 3Y-TZP spectra, the peaks assigned to metal-phosphate derivatives (M-P) and the greater contribution of these peaks on the Co-Cr alloy surface (absorbance scale, 2000–650 cm^−1^ wavenumber range, expanded absorbance scale where indicated).

Representative transmission spectra of the adhesives, used as controls, (“fingerprint” region—expanded 2000–650 cm^−1^ wavenumber range) are presented in Figure 1a. The spectra demonstrated the characteristic peaks of C=O in free (~1720 cm^−1^) or H-bonded status (~1700 cm^−1^), C=C (1635, 945 cm^−1^), C..C (aromatics, 1605, 1590, 800–700 cm^−1^), CH_2_ and CH_3_ (1457–1370 cm^−1^), C-O of ester (1240 cm^−1^), C-O of CH_2_-O (1170 cm^−1^) and the complex contributions of the P=O, P-O, P-O-C, CH_2_-OH, C-O-C at the 1250–900 cm^−1^ band range. The RFTIRM spectra of the polished alloy surface before and after treatments with the adhesives plus solvent rinsing, plotted at the same absorbance scale are illustrated in Figure 1b. The reference alloy surface showed no peaks at the region. After treatment with the adhesives most chemical groups of the original adhesive were found on the alloy surface.

However, there were changes in several peaks such as C=O, with a shift towards the non H-bonded state (1715 cm^−1^), the P=O (1250 cm^−1^) with increased intensity on the alloy surface, the peaks of PO_3_^2−^ (1080, 980 cm^−1^) were reduced in intensity and new peaks appeared at the region 1110–1104 cm^−1^ corresponding to formation of metal-phosphate derivatives (M-P). On several treated alloys, a reduction in the peak intensity of C=C (1636 cm^−1^) over the aromatics (C..C, 1605 cm^−1^) was noticed in comparison with their reference spectra (AD, CB, PO, SB), which implies that the region probed was deficient in aliphatic, but rich in aromatic groups. RFTIRM spectra of zirconia surfaces before and after treatment with the adhesives are depicted in Figure 1c. The reference zirconia surface showed a typical spectrum with a strong absorption of the Zr-O group below 800 cm^−1^. From the surfaces treated with the adhesives, only AB demonstrated clear evidence of M-P formation. Comparison of the peak absorption intensities of the M-P peaks (1115–1110 cm^−1^) identified on the Co-Cr alloy surfaces with the corresponding zirconia revealed a much lower intensity on the latter.

**Figure 2:**
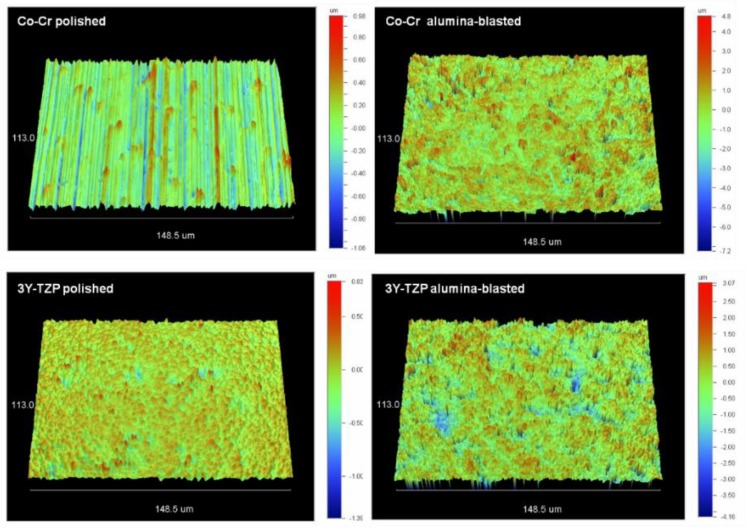
3D-profilometric images of polished and alumina-blasted surfaces of the Co-Cr alloy and 3Y-TZP. Note differences in the z scale bar ranges (40× magnification).

Representative 3D-profilometric images of polished and alumina-blasted surfaces of the Co-Cr alloy and zirconia are illustrated in Figure 2. The results of the roughness parameters tested are summarized in Table 2. Alumina-blasting significantly increased all the roughness parameters for both substrates. The most affected parameter was Sdr demonstrating a 26.5× increase in the alloy and 29× increase in zirconia. The surface to core void volume ratio (Sv/Sc) was reduced by 46% in the alloy and increased by 14% in zirconia. 

**Figure 3:**
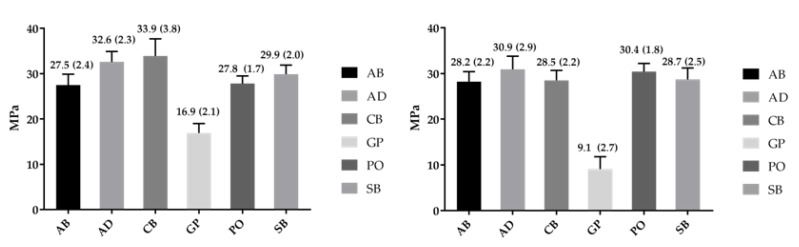
Mean values and standard deviations for shear bond strength to polished Co-Cr alloy before (**left**) and after alumina-blasting (**right**).

**Figure 4:**
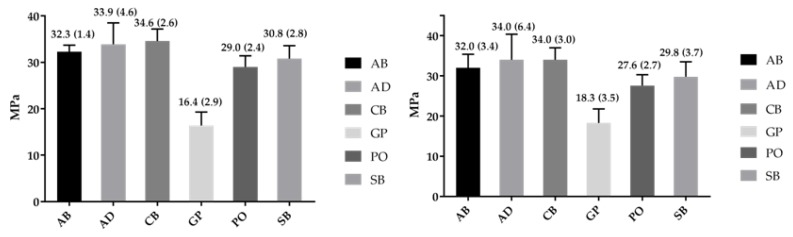
Mean values and standard deviation for shear bond strength to polished 3Y-TZP before (**left**) and after alumina-blasting (**right**).

The mean shear bond strength values and standard deviations are shown in Figure 3 and Figure 4. The results of the Weibull parameters are summarized in Table 3 and Table 4. For polished Co-Cr specimens (Table 3) there was no statistically significant difference in reliability of the adhesives (*β*-parameter, *p* > 0.05), whereas for alumina-blasted Co-Cr, GP showed the lowest *β* values among the adhesives and from the corresponding of polished specimens (*p* < 0.05). The statistical ranking of the characteristic life (*σ*_0_-parameter) of polished specimens was AD, CB > AB, PO, SB > GP. For alumina-blasted specimens the *σ*_0_ ranking was AD > AB > GP, with PO, SB and CB comprising a homogeneous group with no statistically significant differences from AD and AB. Between the two alloy treatment modes (polished and alumina-blasted), GP and PO showed higher *σ*_0_ values on polished specimens. Regarding the *σ*_0.05_ values, the ranking was CB, AD > AB > GP, with SB, PO manifesting no significant differences from CB, AD and AB for polished specimens and SB, AD, CB, PO, AB > GP for alumina-blasted specimens (*p* < 0.05). There was no statistically significant difference between the two treatment modes, except for GP which demonstrated highest values on polished alloy specimens.

For zirconia (Table 4), the ranking in *β* of polished specimens was AB > PO, SB, AD, GP, with CB showing insignificant differences from the adhesives, whereas for alumina-blasted specimens no significant differences in *β* were encountered. The *σ*_0_ rankings were AD, CB > AB > PO > GP (polished) and AD, CB, AB > PO > CP (alumina-blasted), with SB demonstrating insignificant differences from AD, CB, AB (polished) and AD, CB, AB, PO (alumina-blasted), respectively. Assessment of the *σ*_0.05_ values revealed that on polished surfaces the ranking was AB, CB > SB, PO > GP, with AD showing insignificant differences from AB, CB, SB, PO and AB, CB, SB, PO, AD > GP on alumina-blasted. There were no statistically significant differences in *β*, *σ*_0_ and *σ*_0.05_ between polished and alumina-blasted groups (*p* > 0.05). For polished surfaces the *σ*_0.05_ values ranged from 73–85% (Co-Cr) to 64–88% (3Y-TZP) of the *σ*_0_ and for the alumina-blasted 40–88% (Co-Cr) and 59–78% (3Y-TZP) of the *σ*_0_.

**Figure 5:**
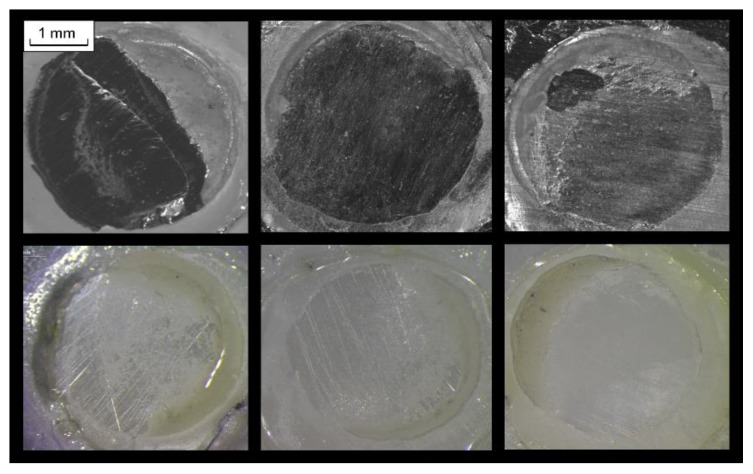
Representative stereomicroscopic images of Co-Cr alloy (**top**) and 3Y-TZP **(bottom**) surfaces after debonding, demonstrating type I (adhesive) and type III (adhesive and resin cohesive) failures with various extent of surface coverage (10× magnification, bar = 1 mm).

Representative debonded alloy and zirconia surfaces are illustrated in Figure 5. The results of the failure mode analysis with the corresponding Chi-square values and probability levels are presented in Table 5. Only type I and III failure modes were identified. There were no statistically significant differences between the failure modes for the same substrate among the adhesives and in the failure modes per adhesive for the two types of treatments performed on each substrate (*p* > 0.05).

## 4. Discussion

Although universal adhesives were introduced for resin bonding to various substrates, the information available for their performance on alloys and zirconia is limited to some bond strength studies. For metals, bonding to stainless-steel and noble alloys has been evaluated [17,19] and SB showed superior bond strength to stainless-steel, while GP to noble alloys. For zirconia, the use of 10-MDP containing adhesives was found to significantly increase the bond strength with resin cements [20] and the bond strength values of restorative composites to zirconia as was mediated by universal adhesives were found higher than of other ceramics [19]. Silica-particle and alumina-particle air-abrasion increased the bond strength of resin composite cements with zirconia, but atlevels insignificantly different from those on polished surfaces [13,21]. In the present study, a Co-Cr alloy and a 3Y-TZP ceramic were chosen as testing substrates. The adhesives were applied on polished and 50 μm alumina-blasted surfaces and a conventional resin composite was used to simulate intraoral repair conditions. Since 10-MDP bonds better to alumina than silica, the former was used for grit-blasting at a pressure and particle size that may induce minimal tetragonal phase destabilization and subsurface damage [22,23]. Alumina-blasting increased all the surface roughness parameters tested including the surface area (Sdr) and the volume retention capacity (Sc), therefore increasing the mechanical retention potential. Zirconia, being a harder substrate was less affected by alumina-blasting than the alloy. 

Although all the adhesives tested contain 10-MDP, which acts as a surfactant and adhesion promoter as a polymerizable monomer, they did not exhibit the same reactivity with the two substrates. The IR analysis showed a more organized adhesive film on the metal substrate than zirconia, with more prominent peaks of adhesive components, despite water and acetone rinsing to remove the loosely bound fractions adsorbed via secondary bonds. Moreover, the peaks assigned to M-P derivatives (1115–1110 cm^−1^) demonstrated higher intensity on the alloy. This may imply a greater physico-chemical affinity of 10-MDP based structures with the Co-Cr alloy. Comparison of the reference transmission FTIR spectra of the adhesives with the RFTIRM spectra obtained on polished surfaces demonstrated reduction in the aliphatic C=C peaks relative to aromatics. This implies that several aliphatic components were rinsed off the surface due to minimal interaction with the substrate (i.e., D3MA, DCDMA). Contrary to this, aromatic peaks were traceable in all spectra, even when aromatic-free adhesive monomers were used, as in the cases of AD, AB, CB, PO and SB. The source of these peaks should be assigned to residual BisGMA. The molecular stiffness and the H-bonding capacity of this monomer attributed to the two aromatic rings and two –OH groups of the monomer backbone structure, may favor adsorption via polar groups onto the substrate after solvent evaporation. Another mechanism of BisGMA adsorption should be through derivatization with acidic monomers and polymers. In this way a stiff crosslinked network may be created after irradiation on the substrate for resin composite bonding. 

On polished Co-Cr specimens, the alloy surface is covered by a passive film composed of various metal oxides such as CoO, MoO_3_ and Cr_2_O_3_, and metallic phases [24]. A more detailed study of the passive oxide zone has documented the presence of a Co^2+^ rich zone at the uppermost oxide film zone of 20–30 Å thickness, and of a Cr^3+^ rich zone at the inner part along with the presence of metallic phases [25]. The superficial oxides are considered to present a co-ordination number of six with –OH and water, whereas the co-ordination number of the inner located oxides is lower. Bonding of acidic groups of adhesive monomers to metal phases is more efficient than to oxides due to the greater availability of free electrons, with metallic Cr and Co demonstrating similar reactivity [25]. From the various oxides formed on the alloy surface those with the lower oxide valence have higher basic character than the oxides with the highest valence [26]. Based on these observations, three bonding mechanisms of carboxyl and phosphate monomers with the Co-Cr alloys have been proposed in descending strength order: ionic bonding with the metallic phase, hydrogen-bonding with the –OH groups of the passive film and dispersion bonding of the hydrated components of the passive layer (mainly oxides) with the polar moieties of the acidic monomers [25].

The alumina-blasted alloy surfaces demonstrated increased roughness as documented by the higher values of amplitude (Sa, Sz), hybrid (Sdr) and functional (Sc, Sv) parameters. The increased surface area (Sdr) combined with the high volume retention capacity (Sc, Sv) create a highly micromechanical retentive surface. The Sv/Sc ratio was considerably reduced after alumina-blasting, apparently due to elimination of the polishing tracks contributing to the deepest surface defects. Moreover, the blasted alloy surface is cleaned from contaminants associated with the preparation process, a new passive layer is spontaneously formed and a fraction of alumina particles is implanted onto material surface, modifying thus the surface chemistry [27]. Whether acidic monomers can bond to these particles is a matter of concern. Phosphate monomers, such as 10-MDP, have demonstrated high and durable resin bond strength to grit-blasted polycrystalline alumina with corundum (α-Al_2_O_3_), but not on polished alumina [28], probably due to increased micromechanical retention and chemical bonding with the activated polycrystalline alumina. The contribution of each individual mechanism is unknown. It has been documented that phosphates interact with corundum at a very slow rate (pH = 5) forming non-protonated bidentate and mono-protonated bidentate surface complexes [29]. It is unclear if such reactions may occur between the phosphate groups of the dental adhesives and the implanted corundum particles on the Co-Cr alloy surface, which are subjected to momentary adhesive treatments and exposed to the unfavorable setting shrinkage vectors of the setting composite. Of particular importance is the area fraction of the loosely bound alumina particles left on the grit-blasted surface, which may affect interfacial bonding. For cementation purposes, removal of this fraction by ultrasonication has been advocated to improve bonding [27], a procedure non-applicable to intraoral repairs. Recently this step has been challenged, suggesting simple air-drying without ultrasonic cleaning as the preferred method to obtain higher early (24 h) bond strength on Co-Cr alloy and zirconia [30], without further addressing the issue of the loosely bound alumina fraction. The results of the present study imply that when light-cured universal dental adhesives were used on the Co-Cr alloy, insignificant differences were obtained in shear bond strength between polished and grit-blasted surfaces.

For the non-etchable zirconia surfaces [14], chemical bonding of carboxyl and phosphate functionalized methacrylate monomers with surface Zr, via hydrogen bonding and ionic interaction has been confirmed [31,32], while thiol and silanol functionalized analogues failed to adsorb on zirconia [32]. In the present study, traceable bonds on zirconia were identified only after AB treatment. This adhesive containing only three monomers demonstrated a very thin absorbent layer on zirconia, improving the sensitivity of the RFTIM in probing reactions at the interface through the adherent film. For the other adhesives, adsorption of chemical groups was verified possibly due to secondary bonding. On grit-blasted surfaces, similar mechanisms with the Co-Cr alloy may apply, regarding the role of the surface implanted alumina particles. However, a major difference is the monoclinic transformation induced in 3Y-TZP by grit-blasting. Such a transformation documented for the same zirconia under the same grit-blasting conditions was in the range of 7.5% [33], and along with the implanted alumina particles, may explain the increase in the Sv/Sc ratio by 14% in alumina-blasted zirconia. The contribution of this “swollen” phase to the interfacial strength is unknown. It has been claimed that the transformed zirconia surface provides increased retention capacity for alumina particles and therefore increases interfacial strength [30]. Nevertheless, such a claim cannot be verified, considering that the monoclinic surface is much weaker than the tetragonal [34]. 

There were differences in the bond strength between the materials tested on the same substrate, despite that all contain 10-MDP as the main surface active and adhesive monomer. These differences may be attributed to several factors including the purity of the raw material [35], presence of co-monomers that may interact with 10-MDP [36], incorporation of many acidic monomers which negatively affect conversion [37], phase separation in systems with immiscible monomers [38], residual solvents in the set material [39] and pH. AD with two phosphate monomers and a carboxylic co-polymer was highly ranked in all the strength parameters tested, reflecting an efficient multi-component approach for bonding with the substrates. On the other hand, CB with a single phosphate monomer proved to be equally efficient, supporting the claims on the critical role of 10-MDP purity in bonding. GP with the lowest pH and evidence of phase separation provided low strength on both substrates and surface treatment modes. This material demonstrated the lowest bond strength on the roughest substrate (alumina-blasted Co-Cr). A possible explanation is that the great amount of solvents applied on rough and non-absorbing substrates, such as metals and polycrystalline ceramics, in conjunction with monomer immiscibility, create surface domains with entrapped solvent and separated monomers into the complex porous structure left after alumina-blasting, reducing thus the solvent effects on surface tension, wettability and viscosity balance requirements for a homogeneous film. The *σ*_0.05_ values for all materials on polished substrate ranged between 64–88% of *σ*_0_, whereas on alumina-blasted from 40–88% of *σ*_0_. Therefore, for alumina-blasted substrates, the early failures in some adhesives seem to occur far below their characteristic life. The failure mode analysis demonstrated mostly type I and a complex type III patterns in many specimens. In type III, residual composite material was mainly located at opposite directions to the loading sites, as a result of the bending stresses developed at the region [40].

A major difference of using universal adhesives on metallic alloys and polycrystalline ceramics rather than on dentin is the lack of fast neutralization of the acidic groups as occurs in contact with dentin mineral phase. Since these adhesives are cured in a separate phase, residual acidity may affect conversion [41] and induce film plasticization by increased water absorption, which further reduces the low mechanical properties of the adhesive films [42]. This may be considered an inherent problem of universal adhesives in comparison with the corresponding metal, zirconia or universal primers used in multistep restorative treatments, where usually a thinner interfacial acidic layer is formed. 

The failure mode analysis demonstrated increased frequency of mixed failure modes in alumina-blasted surfaces with the contribution of the adhesive layer. It seems that the adhesive strength of the film with the substrates was greater than the cohesive strength creating a complex failure pattern on the highly retentive alumina-blasted surfaces, where a thicker film is expected to remain adhered due to the surface topography. Nevertheless, the differences were not statistically significant, supporting previous findings on the lack of correlation between the bond strength results derived from conventional bond strength tests and the mechanical properties of the adhesive films [43]. 

Summarizing the results of the present study it may be concluded that all universal adhesives tested showed evidence of a more prominent affinity with the Co-Cr alloy than with 3Y-TZP. The increase in the surface roughness parameters obtained after alumina-blasting of the polished substrates was not followed by an increase in the shear bond strength. On alloy surfaces, two adhesives demonstrated reduction in their characteristic life after alumina-blasting, whereas on zirconia there was no statistically significant difference between polished and alumina-blasted surfaces. Therefore, the first part of the null hypothesis should be rejected, whereas the second should be accepted. These results may point out important deviations from the performance experienced so far by zirconia- or metal-primers, which demonstrate increased strength on roughened surfaces [44]. It seems that an array of properties associated with the setting capacity of the adhesive film, as previously reviewed, may modify the adhesive potential and the bond strength outcome. The results of the present study should be carefully interpreted for any clinical impact regarding repair procedures with resin composites, for which the experimental design fits better. Conventional rather than micro-shear testing has been chosen to avoid the elaborate sectioning procedure of the hard Cr-Co alloy and zirconia, which could negatively influence the composite-substrate interfacial integrity. In addition, since bonding of the resin composite to the polished substrate was expected to be low, conventional shear testing offers important advantages by reducing the incidence of premature failures [45]. Finally, the bond strength study was limited to a baseline assessment after 1 week of water storage (37 °C), without any further aging, which could reveal the contribution of micromechanical retention after chemical bond degradation [25,26].

## Figures and Tables

**Table 1:** The composition of the universal adhesives tested in the study.

Material	Composition *	Manufacturer
Adhese Universal (AD)	10-MDP, 2-HEMA, BisGMA, MCAP, D3MA, highly dispersed silica, ethanol, water, photoinitiators (pH = 2.8)	Ivoclar-Vivadent, Schaan, Liechtenstein
All-Bond Universal (AB)	10-MDP, 2-HEMA, BisGMA, ethanol, water, photoinitiator (pH = 3.1)	Bisco, Schaumburg, IL, USA
Clearfil Universal Bond (CB)	10-MDP, 2-HEMA, BisGMA, hydrophilic aliphatic dimethacrylate, MPTMS, colloidal silica, photoinitiators (pH = 2.3)	Kuraray Noritake Dental, Okayama, Japan
G-Premio Bond (GP)	10-MDP, 4-MET, MTDP, methacrylic acid ester, silica, acetone, water, photoinitiators (pH = 1.5)	GC Corp., Tokyo, Japan
Prelude One (PO)	10-MDP, Methacryloyloxyalkyl acid carboxylate, 2-HEMA, BisGMA, ethanol (pH = 2.8)	Danville Materials, S. Ramon, CA, USA
Scotchbond Universal Adhesive (SB)	10-MDP, 2-HEMA, BisGMA, DCDMA, MPTMS, VP-copolymer, fumed silica, ethanol, water, photoinitiators (pH = 2.7)	3M ESPE, St. Paul, MN, USA

* According to the manufacturer′s information. 10-MDP: 10-methacryloyloxydecyl dihydrogenphosphate, 2-HEMA: 2-hydroxyethyl methacrylate, BisGMA: Bisphenol-A glycidyl dimethacrylate, MCAP: Methacrylated carboxylic acid polymer, D3MA: Decandiol dimethacrylate, MPTMS: *γ*-methacryloxypropyl trimethoxysilane, 4-MET: 4-methacryloxyethyl trimellitic acid, MDTP: Methacryloyloxydecyl dihydrogen thiophosphate, DCDMA: Decamethylene dimethacrylate, VP-copolymer: Methacrylate-modified polyalkenoic acid copolymer.

**Table 2:** The means and standard deviations of the roughness parameters tested. Same superscript letters show values with no statistically significant difference within each treatment group per parameter (*t*-test, *p* > 0.05). Asterisks denote the Co-Cr (*) and 3Y-TZP (**) pairs where non-parametric analysis was performed (Mann–Whitney test).

Substrate	Treatment	Sa (nm)	Sz (μm)	Sdr (%)	Sc (nm^3^/nm^2^)	Sv (nm^3^/nm^2^)
**Co-Cr**	**Polished**	180 ^a^ (32)	1.7 ^a^ (0.2)	2.9 ^a^ (0.8) *	232.2 ^a^ (23.4)	14.9 ^a^ (2.4) *
	**Alumina Blasted**	652 ^b^ (71) **	5.6 ^b^ (0.5) **	77 ^b^ (5.6) **	822.6 ^b^ (58.9)	98.4 ^b^ (8.7) **
**3Y-TZP**	**Polished**	83 ^a^ (11)	1.2 ^a^ (0.3)	1.5 ^a^ (0.3) *	122.8 ^a^ (10.8)	15.9 ^a^ (4) *
	**Alumina Blasted**	426 ^b^ (58) **	3.9 ^b^ (0.3) **	44 ^b^ (4.7) **	567.8 ^b^ (49.5)	64.8 ^b^ (10.2) **

**Table 3:** The results of the Weibull analysis of the shear bond strength to Co-Cr specimens. Same lowercase superscript letters show values with no statistically significant difference within each row, and same uppercase superscript letters show values with no statistically significant difference for the same parameter within each column (*p* > 0.05).

Co-Cr	Weibull Parameter	AB	AD	CB	GP	PO	SB
**Polished**	**Shape*-β*** **(95% CI)**	12.3 ^a,A^ (7.8–19.3)	18.3 ^a,D^ (11.0–30.3)	16.2 ^a,G^ (9.9–26.7)	9.5 ^a,J^ (5.9–15.3)	17.9 ^a,O^ (11.4–28.0)	16.1 ^a,S^ (10.1–25.6)
	**Scale*-σ*_0_** **(95% CI)/MPa**	28.6 ^a,B^ (27.1–30.2)	33.6 ^b,E^ (32.4–34.8)	35.1 ^b,H^ (33.7–36.5)	17.8 ^c,L^ (16.6–19.2)	28.6 ^a,P^ (27.6–29.7)	30.9 ^a,T^ (29.6–32.3)
	***σ*_0.05_** **(95% CI)/MPa**	22.5 ^a,C^ (19.6–25.8)	28.5 ^b,F^ (25.8–31.5)	29.2 ^b,I^ (26.1–32.6)	13.0 ^c,N^ (10.8–15.7)	24.2 ^a,b,R^ (22.0–26.6)	25.7 ^a,b,U^ (23.1–28.6)
	**r^2^**	0.92	0.95	0.92	0.95	0.85	0.92
**Alumina Blasted**	**Shape*-β*** **(95% CI)**	15.1 ^a,A^ (9.4–24.1)	12.8 ^a,D^ (7.9–20.8)	14.7 ^a,G^ (9.1–23.8)	3.6 ^b,K^ (2.3–5.6)	16.2 ^a,O^ (10.4–25.3)	14.2 ^a,S^ (8.8–23.1)
	**Scale*-σ*_0_** **(95% CI)/MPa**	29.1 ^a,B^ (27.9–30.4)	32.2 ^b,E^ (30.6–33.9)	29.4 ^a,b,H^ (28.2–30.8)	10.0 ^c,M^ (8.4–12.0)	31.3 ^a,b,Q^ (30.0–32.6)	29.7 ^a,b,T^ (28.4–31.1)
	***σ*_0.05_** **(95% CI)/MPa**	23.9 ^a,C^ (21.3–26.8)	25.5 ^a,F^ (22.2–29.3)	24.1 ^a,I^ (21.4–27.1)	4.4 ^b,N^ (2.8–6.9)	24.1 ^a,R^ (21.3–27.3)	26.1 ^a,U^ (23.5–28.9)
	**r^2^**	0.94	0.94	0.92	0.84	0.68	0.94

**Table 4:** The results of the Weibull analysis of the shear bond strength of 3Y-ZTP specimens. Same lowercase superscript letters show values with no statistically significant difference within each row, and same uppercase superscript letters show values with no statistically significant difference for the same parameter within each column (*p* > 0.05).

3Y-TZP	Weibull Parameter	AB	AD	CB	GP	PO	SB
**Polished**	**Shape-β**	30.3 ^a,A^ (17.9–51.4)	10.3 ^b,E^ (6.1–17.4)	16.8 ^a,b,H^ (10.3–27.2)	6.7 ^b,K^ (4.2–10.7)	13.7 ^b,N^ (8.5–21.9)	11.2 ^b,Q^ (7.2–17.5)
	**Scale*-σ*0** **(95% CI)/MPa**	32.9 ^a,C^ (32.3–33.7)	35.8 ^b,F^ (33.8–38.1)	35.7 ^b,I^ (34.4–37.1)	17.5 ^d,L^ (15.9–19.4)	30.0 ^c,O^ (28.6–31.5)	32.0 ^a,b,R^ (30.2–34.0)
	***σ*0.05** **(95%CI)/MPa**	29.9 ^a,D^ (28.1–31.8)	26.8 ^a,b,G^ (22.3–32.1)	29.9 ^a,J^ (26.9–33.2)	11.2 ^c,M^ (8.7–14.8)	24.2 ^b,P^ (21.3–27.5)	24.6 ^b,S^ (21.2–28.5)
	**r2**	0.85	0.96	0.98	0.98	0.91	0.91
**Alumina Blasted**	**Shape-β**	12.2 ^a,B^ (7.4–20.0)	5.6 ^a,E^ (3.6–8.8)	7.63 ^a,H^ (5.02–11.6)	6.8 ^a,K^ (4.0–11.5)	12.0 ^a,N^ (7.5–19.1)	9.0 ^a,Q^ (5.7–14.5)
	**Scale*-σ*0** **(95%CI)/MPa**	33.4 ^a,C^ (31.7–35.3)	36.6 ^a,F^ (32.6–41.2)	36.3 ^a,I^ (33.2–39.5)	19.7 ^b,L^ (17.9–21.7)	28.8 ^c,O^ (27.2–30.4)	31.4 ^a,c,R^ (29.2–33.7)
	***σ*0.05** **(95% CI)/MPa**	26.2 ^a,D^ (22.6–30.3)	21.6 ^a,G^ (16.0–29.1)	24.6 ^a,J^ (19.9–30.3)	12.8 ^b,M^ (9.7–16.7)	22.4 ^a,P^ (19.4–25.9)	22.6 ^a,S^ (18.7–27.3)
	**r2**	0.97	0.91	0.66	0.94	0.93	0.95

**Table 5:** The results of the failure mode analysis.

Treatment	Failure Mode	AB	AD	CB	GP	PO	SB	X^2^	*p*
**Co-Cr Polished**	**Type I**	7	6	7	8	7	6	1.31	0.93
	**Type III**	3	4	3	2	3	4		
**Co-Cr Alumina Blasted**	**Type I**	6	4	5	7	6	5	2.22	0.83
	**Type III**	4	6	5	3	4	5		
**X^2^**		0.22	0.8	0.83	0.27	0.22	0.2		
***p***		0.64	0.37	0.36	0.61	0.64	0.65		
**3Y-TZP Polished**	**Type I**	8	9	9	10	10	9	3.71	0.59
	**Type III**	2	1	1	0	0	1		
**3Y-TZP Alumina Blasted**	**Type I**	7	8	7	10	10	8	6.72	0.24
	**Type III**	3	2	3	0	0	2		
**X^2^**		0.27	0.39	1.25	-	-	0.39		
***p***		0.61	0.53	0.27	-	-	0.53		
